# Antibacterial Activity of New Dibenzoxepinone Oximes with Fluorine and Trifluoromethyl Group Substituents

**DOI:** 10.3390/ijms12106432

**Published:** 2011-09-28

**Authors:** Carmen Limban, Mariana Carmen Chifiriuc

**Affiliations:** 1 Pharmaceutical Chemistry Department, Faculty of Pharmacy, “Carol Davila” University of Medicine and Pharmacy, 6 Traian Vuia, Bucharest 020956, Romania; E-Mail: carmen_limban@yahoo.com; 2 Microbiology Immunology Department, Faculty of Biology, University of Bucharest, Ale. Portocalelor 1-3, Buhcarest 60101, Romania; 3 National Institute of Research and Development for Microbiology and Immunology Cantacuzino, 103 Spl. Independentei, Bucharest 060631, Romania

**Keywords:** dibenz[*b*,*e*]oxepin, methanone derivatives, antibacterial activity, gram-positive, gram-negative

## Abstract

In this paper we present the antimicrobial activity of some newly synthesized dibenz[*b*,*e*]oxepin derivatives bearing the oximino moiety, and fluorine (F) and trifluoromethyl (CF_3_) group substituents. The chemical structure and purity of the new compounds were assessed by using elemental analysis, NMR and FTIR spectroscopy. The new compounds were screened for their antibacterial activity towards Gram-positive and Gram-negative strains, by qualitative and quantitative assays. Our results demonstrated that the CF_3_ and F disubstituted compounds could be considered for the further development of novel antimicrobial drugs.

## 1. Introduction

Dibenz[*b*,*e*]oxepin derivatives represent an interesting class of compounds possessing a wide spectrum of biological and pharmacological activities, such as antidepressant, anxiolytic, anticholinergic and antihistaminic [1,2], antipsychotic [3] and also analgesic, antipyretic and anti-inflammatory properties [4,5]. On the other hand, the oximino moiety has been also reported to have biological significance, being included as a structural fragment in Noxiptilin, an antidepressant drug.

A series of new imidazole derivatives of 6,11-dihydrodibenz[*b*,*e*]oxepines [6] have been proved to exhibit antifungal activity. Leptosphaerin D, an analogue of Arugosin F, is a new polyketide with dibenz[*b*,*e*]oxepin structure, isolated from solid cultures of the ascomycete fungus *Leptosphaeria* sp., which showed antifungal effects against the plant pathogens *Fusarium nivale* and *Piricularia oryzae* [7].

Fluoro and trifluoromethylated compounds are of particular interest as the strong electron-withdrawing effect of F and CF_3_ groups contributes to a number of biologically important molecular properties. Some of the most well known fluorine-containing drugs with antibacterial activity are the fluoroquinolones. The isosteric substitution of hydrogen by fluorine in organofluorines compounds may increase the lipophilicity and thus enhance the rate of cell penetration, which is a very important feature in drug delivery, both referring to prokaryotic, Gram-negative bacteria as well as eukaryotic cells. The higher polarizability due to the C-F bond may give rise to new possibilities for binding to the receptor. Fluorine substitution can also influence pharmacokinetic and pharmacodynamic properties of the molecule [8].

The aim of the present study was to synthesize ({[(*E*/*Z*)-11-dibenz[*b*,*e*]oxepin-11(6*H*)-ylidene]amino}oxy)(substituted-phenyl)methanone derivatives, the phenyl group being disubstituted in different positions with fluorine and trifluoromethyl group, in the purpose to obtain hybrid molecules bearing pharmacophore groups, *i.e.*, dibenz[*b*,*e*]oxepin nucleus, oximino moiety, fluorine and trifluoromethyl group, with improved antimicrobial activity.

## 2. Results and Discussion

Taking into account that, until now, the potential biological activities of different dibenz[*b*,*e*]oxepins derivatives have been tested almost exclusively on eukaryotic cells (*i.e.*, yeasts, molds, protozoan and mammalian cells), our purpose was to assess the *in vitro* antimicrobial activity of dibenz[*b*,*e*]oxepins carrying oximino group at position 11, as well as fluorine and trifluoromethyl groups as substituents, using Gram-positive and Gram-negative bacterial strains, recently isolated from different clinical sources and resistant to beta-lactam antibiotics.

### 2.1. Synthesis and Chemical Characterization

The structure of the new compounds was established on the basis of the elemental analysis and spectral (FTIR and NMR) data.

The new compounds were solid, crystallized, white or light yellow, soluble at normal temperature in acetone, benzene, toluene, xylene, chloroform, dichloromethane and dichloroethane, by heating in inferior alcohols, insoluble in water. The melting points indicated the purity of the new compounds and the elemental analysis closely corresponded to the calculated values.

Structural elucidation of these compounds was also performed by IR and NMR spectra, with all results being in full agreement with the proposed structures.

The IR spectra were given as w—weak band; m—medium band; s—intense band; vs—very intense band and were obtained using the ATR technique. In the IR spectra some significant stretching bands due to υC=N and υN–O were observed at 1597–1611 cm^−1^ and 971–1002 cm^−1^, respectively. The caracteristic bands for the –CH_2_–O– moiety are: νCH_2_ sym: 2865–2897 cm^−1^; νCH_2_ asym: 2962–3001 cm^−1^; δCH_2_ sym: 1304–1310 cm^−1^; δCH_2_ asym: 1480–1482 cm^−1^; νC–O–C sym: 945–988 cm^−1^; νC–O–C asym: 1075–1107 cm^−1^. The IR spectra revealed the presence of –O–C=O absorption bands as νC=O in the region 1740–1764 cm^−1^ and the νC–O at about 1207–1257 cm^−1^. The aromatic rings appeared as ν=C–H in the region 3068–3079 cm^−1^ and νC=C at 1440–1611 cm^−1^. Halogen presence, in the molecules of new compounds, is proved by stretching bands situated at 1001–1098 cm^−1^ (for νC_ar_–F).

The structure of the new compounds is also supported by NMR spectra. The new dibenzoxepins were dissolved in CDCl_3_ and the chemical shifts values, expressed in parts per million (ppm) were referenced downfield to tetramethylsilane, for ^1^H-NMR and ^13^C-NMR and upfield to trifluorocetic acid (δ = −76.20 ppm) for ^19^F-NMR and the coupling constants (J) values in Hertz.

The chemical shifts for hydrogen and carbon atoms were established also by Apt, Gcosy, Ghmqc, Ghmbc experiments.

The ^1^H-NMR data were reported in the following order: chemical shifts, number of protons, multiplicity (s, singlet; d, doublet; dd, double doublet; dq, double quartet; ddd, doublet of double doublets; td, triple doublet; t, triplet; q, quartet, m, multiplet; b, broad), the coupling constants and signal/atom attribution, the signals attribution presenting the major (^M^) and minor (^m^) signals, produced by the *E*/Z isomerism.

The presence of an oxygen atom in the fifth position makes possible the existence of *E*/*Z* isomery, materialized in our spectra through the dedoublation of some of the protons and the carbon signals.

Analyzing the chemical shifts of the dibenz[*b*,*e*]oxepin scaffold in the ^1^H-NMR spectra, CH_2_O protons appeared as a singlet at 5.25–5.26 ppm as minor signal and 5.19–5.20 ppm as major signal.

Also, the ^13^C-NMR spectra displayed the characteristic signal of the suggested structures. The signal at about 165.09–165.88 ppm, is attributed to C-11, the most unscreened carbon atom. The signal of the carbonyl carbon appears in the range of 161.14–165.08 ppm. The C-19 can be found in the range of 121.25–123.46 ppm as quartet. The methylene group (C-6) appears in the range of 70.48–70.61 ppm as major signal and 70.42–70.59 ppm as minor signal the differences between the chemical shifts of the two *E/Z* isomers are insignificant.

### 2.2. Antimicrobial Activity Results

Until now, dibenz[*b*,*e*]oxepins have not been deeply investigated for their antimicrobial activities by other authors. However, there are documented a series of other biological activities for these compounds, demonstrating their interaction with the eukariotic cells, also explaining their antifungal properties [6,7]. During the present study, new dibenzoxepinone oximes bearing fluorinated and trifluoromethylated substituents have been tested for their antimicrobial activity, starting from the hypothesis that the eukariotic cell membrane exhibits similarities with some prokariotic structures (e.g., the outer membrane of the Gram-negative strains). In this purpose, the new compounds have been tested comparatively on Gram-negative and Gram-positive bacterial strains resistant to beta-lactam antibiotics, used in the conventional therapy of this kind of infections.

The qualitative method used for the screening of the antimicrobial activity of the tested compounds indicated only very low diameters of growth inhibition around the paper filter disks, so we did not proceed to measure the diameters of the inhibition zones. These results could be due to the low diffusion rates of the tested substances from the paper filter disks. It must also be mentioned that the intermediary compounds implicated in the synthesis pathway, *i.e.*, 6,11-dihydro-dibenz[*b*,*e*]oxepin-11(6*H*)-one and 11-hydroximino-6,11-dihydro-dibenz[*b*,*e*]oxepin have been also screened by disk diffusion, but no inhibition of the microbial growth was observed.

**Figure 1:**
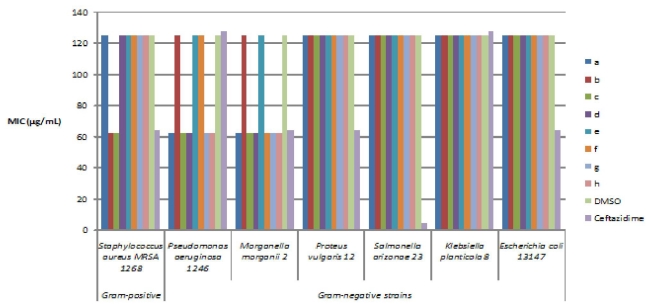
Minimum inhibitory concentration (MIC) of dibenz[*b*,*e*]oxepins.

The results of the quantitative assay of the antimicrobial activity, expressed by the MIC values, of the new compounds are summarized in Figure 1.

The quantitative assay revealed that out of the tested compounds, all were more active that ceftazidime, exhibiting lower MIC values than the antibioticon *Klebsiella planticola*, six on *Morganella morganii*, five on *Pseudomonas aeruginosa* and two on methicillin resistant *Staphylococcus aureus* (Figure 1). A MIC value superior to 250 μg/mL was considered as corresponding to a low, between 250 μg/mL and 125 μg/mL to moderate and under 62.5 μg/mL to a good antimicrobial activity.

In general, the positions of fluorine and trifluoromethyl substituents on phenyl ring significantly influenced the antibacterial activity. It is already known that fluorine could exhibit an intrinsic microbicidal activity. Insertion of fluorine in a strategic position of a molecule has emerged as a very powerful and versatile tool for the development of the compounds endowed with biological activities. The presence of fluorine often leads to increased lipid solubility, thereby enhancing the rate of *in vivo* absorption and transport of drugs [9,10].

The most active compound, concerning both the intensity of the antimicrobial activity and the microbial spectrum proved to be ({[(*E*/*Z*)-11-dibenz[*b*,*e*]oxepin-11(6*H*)-ylidene]amino}oxy) [2-fluoro-5-(trifluoromethyl)-phenyl]methanone (c), which has been more active than ceftazidime on methicillin resistant *Staphylococcus aureus*, *Pseudomonas aeruginosa* and *Morganella morganii*. It could be concluded that the substitution with F in position 2 and CF_3_ in 5 is the most favorable for the antimicrobial activity. Interestingly, the reversion of the two subtituents (F in 5 and CF_3_ in 2) leads to the loss of the anti-*Staphylococcus* activity.

The 2-F, 4-CF_3_ (b) and the 2-F, 5-CF_3_ (c) disubstituted derivatives exhibited good antibacterial activity, superior to ceftazidime and DMSO against *Staphylococcus aureus*.

The lowest antimicrobial activity was observed for the substitutions in 3 and 5 positions (e). The substitutions in the positions 2 and 4 (b) or 3 and 5 (e) are not favorable for the antimicrobial activity against *Morganella morganii* and *Pseudomonas aeruginosa*.

For *Proteus vulgaris*, *Salmonella arizonae* and *Escherichia coli*, the activity of the tested compounds was similar to that of the DMSO solvent and inferior to the control antibiotic. For *Klebsiella planticola*, all tested substances showed similar MIC values as for the DMSO and lower than the tested antibiotic.

Although the differences between DMSO and the tested compounds did not exceed one dilution, it must be noticed that in some cases, our compounds were more active than ceftazidime. The relatively high MIC values could be due, on the other hand, to the fact that the bacterial strains chosen to be tested were of clinical origin and resistant to antibiotics.

## 3. Experimental Section

### 3.1. Chemicals and Methods

All chemicals and solvents were purchased from Merck, Fluka and Sigma-Aldrich and were of commercial quality. They were used as received, except 1,2-dichloroethane (anhydrized over calcium chloride and distilled at normal pressure), pyridine (stored over potassium hydroxide and then distilled), and benzene (kept overnight with sodium and then distilled).

Melting points were uncorrected and determined in open capillary tubes on an Electrothermal 9100 apparatus.

Elemental analyses were performed on a Perkin Elmer CHNS/O Analyser Series II 2400 apparatus, the results being within ±0.4% of the theoretical values.

IR spectra were recorded on a FT-IR Bruker Vertex 70 spectrophotometer.

NMR spectra were recorded on a Varian Unity Inova 400 instrument operating at 400 MHz for ^1^H, 100 MHz for ^13^C and 376.279 MHz for ^19^F-NMR.

#### 3.1.1. Synthesis of the New Compounds

The dibenz[*b*,*e*]oxepins were synthesized according to the previusly described procedure [11]. The 2-phenoxymethylbenzoic acid was prepared in the first stage, by refluxing the phtalide with potassium phenoxyde in xylene. This gives the 2-phenoxymethylbenzoic acid potassium salt, which has good solubility in potassium hydroxide aqueous solution allowing its facile separation from xylene. The aforementioned acid was then precipitated using a mineral acid solution. The potassium salt of phenol was obtained using phenol and potassium hydroxide in xylene, the resulting water being removed by azeotropic distillation. The 6,11-dihydro-dibenz[*b*,*e*]oxepin-11(6*H*)-one was synthesized in the second stage by a Friedel-Crafts cyclization of the 2-phenoxymethylbenzoic acid chloride in dry 1,2-dichloroethane. The acid chloride was obtained by refluxing the corresponding acid with thionyl chloride and anhydrous 1,2-dichloroethane as reaction medium. The new compounds were prepared by acylation of the 11-hydroximino-6,11-dihydro-dibenz[*b*,*e*]oxepin with various substituted benzoic acid chlorides, in dry benzene and in the presence of anhydrous pyridine as a proton fixator. The resulting solid was recrystallized from isopropanol to yield the new *O*-acyl-oximino-dibenz[*b*,*e*]oxepins. The oxime was obtained by treating the 6,11-dihydro-dibenz[*b*,*e*]oxepin-11(6*H*)-one with hydroxylamine hydrochloride in presence of pyridine. The chemical structure of the new compounds is presented in Table 1.

##### 3.1.1.1. ({[(*E*/*Z*)-11-dibenz[*b*,*e*]oxepin-11(6*H*)-ylidene]amino}oxy)[2-fluoro-3-(trifluoromethyl)- phenyl]methanone (a) (*E*/*Z* isomers mixture in ratio 1:2.45 estimated by ^1^H-NMR)

**FT-IR** (solid in ATR, ν cm^−1^): 532 (w), 572 (w), 630 (w), 705 (m), 748.(vs), 827 (m), 865 (m), 948 (w), 979 (m), 1075 (s), 1098 (s), 1122 (vs), 1159 (m), 1195 (s), 1237 (m), 1269 (s), 1305 (m), 1333 (s), 1443 (m), 1462 (s), 1481 (w), 1595 (m), 1611 (m), 1740 (vs), 2987 (w), 3072 (w), 3104 (w).

**^19^F-NMR** (CDCl_3_): δ-61.97(3F, d, ^4^*J*_(F-15–F-14)_ = 13.78 Hz, CF_3_), δ-109.60 (1F, q, ^4^*J*_(F-15–F-14)_ = 13.78 Hz, F-14).

**^1^H-NMR** (CDCl_3_): δ 5.19 (s, H-6 ^M^), 5.25 (s, H-6 ^m^), 6.91 (dd, *J* = 1.1, 8.4 Hz, H-4 ^M^), 6.98 (dd, *J* = 1.1, 8.4 Hz, H-4 ^m^), 7.02 (ddd, *J* = 1.1, 7.0, 8.0 Hz, H-2 ^M^), 7.03 (ddd, *J* = 1.1, 7.0, 8.0 Hz, H-2 ^m^), 7.29 (1H, m, *J*_(H-17–H-16)_= 8.2 Hz, H-17), 7.36 (1H, ddd, *J* = 1.7, 7.2, 8.4 Hz, H-3), 7.49–7.40 (3H, m, H-7, H-8, H-9), 7.61 (m, H-10 ^M^), 7.66 (m, H-10 ^m^), 7.83–7.73 (m, H-16, H-1 ^m^), 7.87 (dd, *J* = 1.8, 8.0 Hz, H-1 ^M^), 8.06 (ddd, *J*_(F-H-18)_= 6.5 Hz, *J*_(H-17–H-18)_= 8.2 Hz, *J*_(H-18–H-16)_= 1.7 Hz, H-18 ^M^), 8.13 (ddd, *J*_(F-H-18)_= 6.5 Hz, *J*_(H-17–H-18)_= 8.2 Hz, *J*_(H-18–H-16)_= 1.7 Hz, H-18 ^m^).

**^13^C-NMR** (CDCl_3_): δ 70.4 (C-6 ^m^), 70.5 (C-6 ^M^), 118.4, 119.2, 119.4, 120.0 ^M^, 120.4 ^m^, 120.8 ^m^, 121.5 ^M^, 123.3 (q, *J*_(F-C)_= 271.1 Hz, C-19), 124.1 (d, ^3^*J*_(F-C)_ = 4.4 Hz, C-18), 127.2, 127.9 ^m^, 128.3 ^M^, 128.4 ^m^, 128.5 ^M^, 129.3 ^m^, 130.7 ^M^, 130.9 ^M^, 131.0 ^m^, 131.7 (m, C-15), 132.6, 132.8 ^m^, 132.9 ^M^, 134.2 (C-10a ^m^), 134.2 (C-10a ^M^), 136.0 (C-16 ^M^), 136.1 (C-16 ^m^), 155.8 (C-4a ^m^), 157.6 (C-4a ^M^), 162.1 (d, *J*_(F-C)_ = 252.7 Hz, C-14), 165.4 (C-11), 162.7 (C-12).

##### 3.1.1.2. ({[(*E*/*Z*)-11-dibenz[*b*,*e*]oxepin-11(6*H*)-ylidene]amino}oxy)[2-fluoro-4-(trifluoromethyl)-phenyl]methanone (b) (*E*/*Z* isomers mixture in ratio 1:4.3 estimated by ^1^H-NMR)

**FT-IR** (solid in ATR, ν cm^−1^): 545 (w), 602 (w), 634 (w), 698 (w), 756 (s), 835 (w), 882 (m), 916 (w), 977 (s), 1007 (m), 1049 (m), 1084 (vs), 1127 (s), 1176 (s), 1214 (s), 1236 (m), 1277 (m), 1304 (m), 1332(s), 1430 (s), 1481 (w), 1507 (w), 1606 (m), 1745 (vs), 2865 (w), 2917 (w), 2993 (w), 3071 (w).

**^19^F-NMR** (CDCl_3_): δ-63.90 (3F, bs, CF_3_), δ-105.11 (1F, bs, F-14).

**^1^H-NMR** (CDCl_3_): δ 5.19 (s, H-6 ^M^), 5.25 (s, H-6 ^m^), 6.91 (dd, *J* = 1.1, 8.4 Hz, H-4 ^M^), 6.98 (dd, *J* = 1.1, 8.4 Hz, H-4 ^m^), 7.02 (1H, td, *J* = 8.4, 1.1 Hz, H-2), 7.51–7.33 (6H, m, H-3, H-7, H-8, H-9, H-15, H-18), 7.59 (1H, m, H-10), 7.88 (1H, dd, *J* = 1.8, 8.0 Hz, H-1), 7.99 (bt, ^5^*J*_(H-17–F-14)_ = 7.2 Hz, ^3^*J*_(H-17–H-18)_ = 7.2 Hz, H-17 ^M^), 8.08 (bt, ^5^*J*_(H-17–F-14)_ = 7.2 Hz, ^3^*J*_(H-17–H-18)_ = 7.2 Hz, H-17 ^m^).

**^13^C-NMR** (CDCl_3_): δ 70.4 (C-6 ^m^), 70.5 (C-6 ^M^), 114.7 (dq, *J*_(F-C-15)_ = 25.6 Hz, *J*_(F3-C-15)_ = 3.8 Hz, C-15), 119.4 (C-1 ^a^), 120.0, 121.0 (dq, C-17), 121.5, 123.5 (q, *J*_(F-C)_ = 272.3 Hz, C-19), 127.2 ^M^, 127.9 ^m^, 128.3 (d, *J*_(F-C)_ = 2.2 Hz, C-18), 128.4 ^M^, 128.5 ^m^, 130.6, 130.9, 132.7, 132.9, 133.2, 134.3, 136.5, 136.8, 157.6 (C-4 ^a^), 162.6 (C-11), 162.7 (d, *J*_(F-C)_ = 245.8 Hz, C-14), 165.5 (C-12).

##### 3.1.1.3. ({[(*E*/*Z*)-11-dibenz[*b*,*e*]oxepin-11(6*H*)-ylidene]amino}oxy)[2-fluoro-5-(trifluoromethyl)- phenyl]methanone (c) (*E*/*Z* isomers mixture in ratio 1:2.2 estimated by ^1^H-NMR)

**FT-IR** (solid in ATR, ν cm^−1^): 548 (w), 616 (w), 681 (w), 753 (s), 794 (w), 847 (m), 895 (m), 977 (s), 1011 (m), 1038 (vs), 1078 (s), 1110 (s), 1161 (m), 1207 (vs), 1271 (w), 1310 (m), 1334 (s), 1445 (m), 1483 (w), 1606 (m), 1764 (vs), 2883 (w), 2986 (w), 3070 (w).

**^19^F-NMR** (CDCl_3_): δ-62.81 (3F, bs, CF_3_), δ-102.18 (1F, bs, F-14).

**^1^H-NMR** (CDCl_3_): δ 5.19 (s, H-6 ^M^), 5.25(s, H-6 ^m^), 6.91 (dd, *J* = 1.1, 8.4 Hz, H-4^M^), 7.06–6.97 (m, H-2, H-4 ^m^), 7.23 (t, *J* = 7.8 Hz, H-15 ^M^), 7.28 (t, *J* = 7.8 Hz, H-15 ^m^), 7.51–7.32 (5H, m, H-2, H-3, H-7, H-8, H-9), 7.60 (bd, *J* = 7.8 Hz, H-16 ^M^), 7.76 (bd, 7.8 Hz, H-16 ^m^), 7.77 (1H, m, H-10), 7.88 (1H, dd, *J* = 1.8, 8.0 Hz, H-1), 8.25 (1H, bd, *J* = 4.9 Hz, H-18).

**^13^C-NMR** (CDCl_3_): δ 70.5 (C-6 ^m^), 70.6 (C-6 ^M^), 118.1 (m, C-15), 119.4(C-1a), 120.0 ^M^, 120.5 ^m^, 120.8 ^M^, 121.5 ^m^, 123.1 (q, *J*_(F-C-19)_ = 270.9 Hz, C-19), 127.2 ^m^, 127.9 ^M^, 128.2 ^M^, 128.5 ^m^, 128.4 ^M^, 128.5 ^m^, 129.4, 130.0 (m, C-18), 130.6 ^M^, 130.7 ^m^, 130.9, 131.9 (m, C-16), 132.7 ^M^, 132.9 ^m^, 134.2 (C-10a ^m^), 134.3 (C-10a ^M^), 136.7, 157.6 (C-4a), 160.3 (q, *J*_(CF3-C-17)_ = 4.4 Hz, C-17), 163.5 (d, *J*_(F-C)_ = 264.4 Hz, C-14), 164.53 (C-11), 165.4 (C-12).

##### 3.1.1.4. ({[(*E*/*Z*)-11-dibenz[*b*,*e*]oxepin-11(6*H*)-ylidene]amino}oxy)[3-fluoro-4-(trifluoromethyl)- phenyl]methanone (d) (*E*/*Z* isomers mixture in ratio 1:2.4 estimated by ^1^H-NMR)

**FT-IR** (solid in ATR, ν cm^−1^): 561 (w), 591 (w), 609 (w), 641 (w), 673 (w), 705 (w), 727 (m), 754 (s), 774 (m), 796 (w), 824 (m), 843 (w), 878 (m), 936 (s), 988 (m), 1004 (m), 1045 (s), 1085 (vs), 1123 (s), 1139 (vs), 1192 (vs), 1222 (m), 1250 (vs), 1281 (m), 1312 (s), 1328 (s), 1378 (w), 1418 (s), 1443 (m), 1482 (m), 1506 (w), 1583 (m), 1605 (m), 1630 (w), 1761 (vs), 2892 (w), 2965 (w), 3068 (w).

**^19^F-NMR** (CDCl_3_): δ-62.34 (3F, d, ^4^*J*_(3F-16–F-15)_ = 12.64 Hz, CF_3_), δ-112.94 (1F, q, ^4^*J*_(3F-16–F-15)_ = 12.64 Hz, F-15).

**^1^H-NMR** (CDCl_3_): δ 5.20 (s, H-6 ^M^), 5.26 (s, H-6 ^m^), 6.92 (dd, *J* = 1.2, 8.4 Hz, H-4 ^M^), 7.03 (dd, *J* = 1.2, 8.4 Hz, H-4 ^m^), 7.03 (ddd; *J* = 1.2, 7.2, 8.2 Hz, H-2 ^M^), 7.08 (ddd, *J* = 1.2, 7.2, 8.2 Hz, H-2 ^m^), 7.37 (1H, ddd, *J* = 1.4, 7.2, 8.2 Hz, H-3), 7.58–7.33 (4H, m, H-7-, H-8, H-9, H-10), 7.84–7.64 (m, H-1 ^m^, H-14, H-17, H-18), 7.89 (dd, *J* = 1.8, 8.0 Hz, H-1 ^M^).

**^13^C-NMR** (CDCl_3_): δ 70.5 (C-6 ^m^), 70.6 (C-6 ^M^), 118.1 (d, *J*_(F-C)_ = 22.7 Hz, C-14 ^M^), 118.3 (d, *J*_(F-C)_ = 22.7 Hz, C-14 ^m,^), 119.2 (C-1a), 120.1, 121.6, 122.0 (q, *J*_(F-C-19)_ = 271.6 Hz, C-19), 125.3 (d, *J*_(F-C-13)_ = 4.3 Hz, C-13 ^M^), 125.4 (d, *J*_(F-C-13)_ = 4.3 Hz, C-13 ^m^), 127.2 ^m^, 127.6, 127.8 ^M^, 127.9 ^m^, 128.5 ^M^, 128.8 ^M^, 129.4 ^m^, 130.3, 130.8 (d, *J*_(F-C-18)_= 2.8 Hz, C-18), 132.9 ^m^, 133.0 ^M^, 134.1 (C-10a ^m^), 134.2 (C-10a ^M^), 134.5, 136.7, 156.0 (C-4a ^m^), 157.7 (C-4a ^M^), 160.3 (q, *J*_(CF3-C-16)_ = 4.4 Hz, C-16), 159.6 (d, *J*_(F-C-15)_ = 254.8 Hz, C-15), 164.6 (C-11), 165.5 (C-12).

##### 3.1.1.5. ({[(*E*/*Z*)-11-dibenz[*b*,*e*]oxepin-11(6*H*)-ylidene]amino}oxy)[3-fluoro-5-(trifluoromethyl)-phenyl]methanone (e) (*E*/*Z* isomers mixture in ratio 1:4.4 estimated by ^1^H-NMR)

**FT-IR** (solid in ATR, ν cm^−1^): 554 (w), 591 (w), 628 (w), 641 (w), 689 (m), 706 (w), 755 (s), 783 (w), 807 (w), 885 (s), 921 (w), 945 (m), 1002 (m), 1044 (w), 1103 (m), 1137 (vs), 1181 (vs), 1231 (vs), 1250 (s), 1336 (m), 1352 (m), 1443 (w), 1482 (m), 1607 (m), 1763 (s), 3001 (w), 3079 (w), 3081 (w).

**^19^F-NMR** (CDCl_3_): δ-63.51 (3F, bs, CF_3_), δ-109.19 (1F, bs, F-15).

**^1^H-NMR** (CDCl_3_): δ 5.20 (s, H-6 ^M^), 5.26 (s, H-6 ^m^), 6.92 (dd, *J* = 1.2, 8.4 Hz, H-4 ^M^), 7.03 (dd, *J* = 1.2, 8.4 Hz, H-4 ^m^), 7.03 (ddd, *J* = 8.2, 7.2, 1.2 Hz, H-2 ^M^), 7.09 (ddd, *J* = 8.2, 7.2, 1.2 Hz, H-2 ^m^), 7.37 (1H, ddd, *J* = 1.4, 7.2, 8.2 Hz, H-3), 7.61–7.34 (m, H-7, H-8, H-9, H-10 ^m^, H-14), 7.68 (m, H-10 ^M^), 7.79 (1H, ddd, *J* = 2.4, 1.8 Hz, *J*_(F-H-16)_ = 8.6 Hz, H-16), 7.89 (1H, dd, *J* = 1.8, 8.0 Hz, H-1), 7.92 (bs, H-18 ^M^), 8.40 (bs, H-18 ^m^).

**^13^C-NMR** (CDCl_3_): δ 70.6 (C-6 ^m^), 70.6 (C-6^M^), 117.6 (dq, *J*_(F-C-16)_ = 22.7.2 Hz, *J*_(F3-C-16)_ = 3.7Hz, C-16), 119.2 (C-1a), 120.1, 121.6, 121.8 (q, *J*_(F-C-19)_ = 269.8 Hz, C-19), 122.4 (q, *J*_(F-C)_ = 3.6 Hz, C-18), 123.0, 127.8, 128.5, 128.8, 130.3, 130.8, 131.9, 132.0 (d, *J*_(F-C)_ = 8.0 Hz, C-13), 132.9, 133.0, 134.2 (C-10a), 157.7 (C-4a), 161.1 (C-11), 162.3 (d, *J*_(F-C-15)_ = 250.0 Hz, C-15), 165.5 (C-12).

##### 3.1.1.6. ({[(*E*/*Z*)-11-dibenz[*b*,*e*]oxepin-11(6*H*)-ylidene]amino}oxy)[4-fluoro-2-(trifluoromethyl)- phenyl]methanone (f) (*E*/*Z* isomers mixture in ratio 1:3 estimated by ^1^H-NMR)

**FT-IR** (solid in ATR, ν cm^−1^): 547 (w), 579 (w), 632 (m), 688 (w), 753 (s), 878 (m), 910 (m), 971 (s), 1001 (m), 1037 (s), 1082 (s), 1139 (vs), 1246 (vs), 1310 (s), 1431 (m), 1480 (m), 1604 (m), 1761 (vs), 2888 (w), 2990 (w), 3071 (w).

**^19^F-NMR** (CDCl_3_): δ-60.30 (3F, d, ^5^*J*_(F-16–3F-19)_ = 5.74 Hz, CF_3_), δ-105.30 (1F, q, ^4^*J*_(3F-19–F-16)_ = 5.74 Hz, F-16).

**^1^H-NMR** (CDCl_3_): δ 5.19 (s, H-6 ^M^), 5.25 (s, H-6 ^m^), 6.90 (dd, *J* = 1.4, 8.2 Hz, H-4 ^M^), 6.93 (td, *J* = 8.2, 1.4 Hz, H-2 ^m^), 6.97 (dd, *J* = 1.4, 8.2 Hz, H-4 ^m^), 7.01 (td, *J* = 8.2, 1.4 Hz, H-2 ^M^), 7.22–7.49 (7H, m, H-3, H-7, H-8, H-9, H-10, H-15, H-17), 7.69 (dd, *J*_(F-H-18)_ = 5.4 Hz, *J*_(H-18-H-17)_ = 8.6 Hz, H-18 ^M^), 7.81 (dd, *J*_(F-H-18)_ = 5.4 Hz, *J*_(H-18-H-17)_ = 8.6 Hz, H-18 ^m^), 7.54 (dd, *J* = 1.8, 8.0 Hz, H-1 ^m^), 7.87 (dd, *J* = 1.8, 8.0 Hz, H-1 ^M^).

**^13^C-NMR** (CDCl_3_): δ 70.4 (C-6 ^m^), 70.48 (C-6 ^M^), 115.0 (dq, *J*_(F-C-15)_= 25.6 Hz, *J*_(F3-C-15)_= 5.1 Hz, C-15), 118.8 (*J*_(F-C-17)_= 21.2 Hz, C-17 ^M^), 118.8 (*J*_(F-C-17)_ = 21.2 Hz, C-17 ^m^), 119.3 (C-1a), 120.0 ^M^, 120.5 ^m^, 120.8 ^m^, 121.5 ^M^, 122.2 (q, *J*_(F3-C-19)_ = 264.3 Hz, C-19), 127.7 ^M^, 127.9 ^m^, 128.4^M^, 128.5 ^m^, 129.4, 130.5, 130.5 ^M^, 130.7 ^m^, 130.8, 132.7, 132.9 (dq, *J*_(F-C-14)_= 12.4 Hz, *J*_(F3-C-14)_ = 8 Hz, C-14), 132.9, 134.0 (C-10a ^m^), 134.3 (C-10a ^M^), 136.7, 155.8 (C-4a ^m^), 157.5 (C-4a ^M^), 162.9 (C-11), 163.8 (d, *J*_(F-C-16)_ = 253.3 Hz, C-16), 164.6 (C-12 ^M^), 165.6 (C-12 ^m^).

##### 3.1.1.7. ({[(*E*/*Z*)-11-dibenz[*b*,*e*]oxepin-11(6*H*)-ylidene]amino}oxy)[4-fluoro-3-(trifluoromethyl)-phenyl]methanone (g) (*E*/*Z* isomers mixture in ratio 1:4.8 estimated by ^1^H-NMR)

**FT-IR** (solid in ATR, ν cm^−1^): 548 (w), 631 (w), 675 (w), 707 (w), 731 (w), 752 (s), 846 (m), 981 (m), 1006 (w), 1051 (m), 1077 (s), 1108 (w), 1133 (m), 1147 (s), 1161 (s), 1223 (s), 1243 (s), 1269 (m), 1305 (m), 1325 (s), 1423 (m), 1441 (m), 1464 (w), 1480 (w), 1601 (m), 1759 (vs), 2897 (w), 2962 (w), 3073 (w).

**^19^F-NMR** (CDCl_3_): δ-62.24(3F, d, ^4^*J*_(3F-15–F-16)_ = 12.06 Hz, CF_3_), δ-106.47(1F, q, ^4^*J*_(3F-15–F-16)_ = 12.06 Hz, F-16).

**^1^H-NMR** (CDCl_3_): δ 5.20 (s, H-6 ^M^), 5.26 (s, H-6 ^m^), 6.92 (dd, *J* = 1.4, 8.2 Hz, H-4 ^M^), 7.03 (dd, *J* = 1.4, 8.2 Hz, H-4 ^m^), 7.04 (td, *J* = 8.2, 1.4 Hz, H-2 ^M^), 7.08 (td, *J* = 8.2, 1.4 Hz, H-2 ^m^), 7.26 (dd, *J*_(H-17-H-18)_ = 8.2 Hz, *J*_(F-H-17)_= 10.4 Hz, H-17 ^M^), 7.29 (dd, *J*_(H-17–H-18)_ = 8.2 Hz, *J*_(F-H-17)_ = 10.4 Hz, H-17 ^m^), 7.37 (1H, ddd, *J* = 1.7, 7.2, 8.0 Hz, H-3), 7.57–7.34 (3H, m, H-7, H-8, H-9), 7.68 (dq, *J*_(F-H-14)_ = 8.0 Hz, *J*_(F3-H-14)_ = 1.8 Hz, H-14 ^M^), 7.89 (1H, dd, *J* = 1.8, 8.0 Hz, H-1), 8.18–8.10 (m, H-10 ^M^, H-18), 8.24 (m, H-14 ^m^), 8.30 (m, H-10 ^m^).

**^13^C-NMR** (CDCl_3_): δ 70.5 (C-6 ^m^), 70.6 (C-6 ^M^), 117.6 (d, *J*_(F3-C-17)_ = 21.2 Hz, C-17), 119.3 (C-1a), 120.0 (C-4), 121.6 (C-2), 122.0 (q, *J*_(F3-C-19)_ = 270.3 Hz, C-19), 125.3 (d, *J*_(F-C-13)_ = 2.9 Hz, C-13), 127.8 (C-10 ^M^), 127.9 (C-10 ^m^), 128.4, 128.7, 129.3 (m, C-15), 130.4, 130.7 (C-1 ^m^), 130.8, 130.8 (C-1 ^M^), 132.8 (C-3 ^m^), 132.9 (C-3 ^M^), 133.0, 134.3 (C-10a), 135.8 (d, *J*_(F-C)_ = 9.5 Hz, C-14), 157.6 (C-4a), 161.4 (C-11), 162.7 (d, *J*_(F-C-16)_ = 263.6 Hz, C-16), 166.0 (C-12).

##### 3.1.1.8. ({[(*E*/*Z*)-11-dibenz[*b*,*e*]oxepin-11(6*H*)-ylidene]amino}oxy)[5-fluoro-2-(trifluoromethyl)-phenyl]methanone (h) (*E*/*Z* isomers mixture in ratio 1:2.8 estimated by ^1^H-NMR)

**FT-IR**(solid in ATR, ν cm^−1^): 518 (w), 591 (m), 630 (w), 680 (w), 757 (s), 835 (m), 885 (m), 932 (m), 984 (m), 1031 (s), 1076 (s), 1115 (vs), 1153 (vs), 1196 (s), 1257 (s), 1305 (vs), 1440 (w), 1480 (w), 1597 (m), 1759 (vs), 2875 (w), 3078 (w)

**^19^F-NMR** (CDCl_3_): δ-59.25 (3F, bs, CF_3_); δ-106.49 (1F, bs, F-14).

**^1^H-NMR**(CDCl_3_): δ 5.19 (s, H-6 ^M^), 5.25 (s, H-6 ^m^), 6.90 (dd, *J* = 1.4, 8.2 Hz, H-4 ^M^), 6.94 (td, *J* = 8.2, 1.4, H-2 ^m^), 6.97 (dd, *J* = 1.4, 8.2 Hz, H-4 ^m^), 7.02 (td, *J* = 8.2, 1.4 Hz, H-2 ^M^), 7.23–7.49 (6*H*, m, H-3, H-7, H-8, H-9, H-16, H-18), 7.54 (dd, *J* = 1.8, 8.0 Hz, H-1 ^m^), 7.66 (m, H-10 ^m^), 7.72 (dd, *J*_(F-H-15)_ = 5.1 Hz, *J*_(H-15-H-16)_ = 8.8 Hz, H-15 ^M^), 7.76(dd, *J*_(F-H-15)_ = 5.1 Hz, *J*_(H-15–H-16)_ = 8.8 Hz, H-15 ^m^), 7.86 (dd, *J* = 1.8, 8.0 Hz, H-1 ^M^).

**^13^C-NMR** (CDCl_3_, ppm): δ 70.4 (C-6 ^m^), 70.5 (C-6 ^M^), 117.7 (d, *J*_(C-F)_ = 24.9 Hz, C-18 ^M^), 117.7 (d, *J*_(C-F)_= 24.9 Hz, C-18 ^m^), 118.4 (d, *J*_(C-16-F)_ = 22.0 Hz, C-16 ^M^), 118.4 (d, *J*_(C-16-F)_ = 22.0 Hz, C-16 ^m^), 119.5 (C-1a), 120.6, 120.9, 121.5, 127.1, 127.7, 127.9, 128.4, 129.6, 129.5 (dq, *J*_(F-C-14)_ = 3.8 Hz, *J*_(F3-C-14)_ = 5.1 Hz, C-14), 130.4 ^m^, 130.6 ^M^, 130.7 ^m^, 130.8 ^M^, 132.7, 132.7 ^m^, 132.9 ^M^, 134.1 (C-10a ^m^), 134.2 (C-10a ^M^), 155.8 (C-4a ^m^), 157.6 (C-4a ^M^), 163.9 (d, *J*_(F-C-17)_ = 241.6 Hz, C-17), 165.1 (C-11), 165.9 (C-12).

### 3.2. Microbiological Assays

#### 3.2.1. Microbial Strains

The antimicrobial activity of the investigated compounds was tested against the following bacterial strains: Gram-positive, coagulase-positive, methicillin resistant *Staphylococcus aureus* 1268 and Gram-negative, beta-lactam resistant *Pseudomonas aeruginosa* 1246, *Morganella morganii* 2, *Proteus vulgaris* 12, *Salmonella arizonae* 23, *Klebsiella planticola* 8 and *Escherichia coli* 13147 strains.

The coagulase-positive, methicillin resistant *Staphylococcus aureus* and the *Pseudomonas aeruginosa* strain were recently isolated from central venous catheters associated infections, while the *Enterobacteriaceae* strains were isolated from different cases of acute diffuse peritonitis.

All tested strains were identified by VITEK I automatic system. VITEK cards for identification and susceptibility testing (GNS-522) were inoculated and incubated according to the manufacturer’s recommendations. The results were interpreted by using software version AMS R091. Bacterial suspensions of density corresponding to 0.5 McFarland UI obtained from 18 h bacterial cultures developed on solid media were used in the experiments. The antimicrobial activity of the new compounds was tested on Mueller-Hinton agar medium.

#### 3.2.2. Stock Solutions

The tested compounds were solubilised in DMSO and used for the antimicrobial activity screening at 1 mg/ mL concentration.

#### 3.2.3. Qualitative Screening of the Antimicrobial Properties of the Tested Compounds

The qualitative screening was performed by an adapted disk diffusion method. In this purpose, Petri dishes with Mueller Hinton medium were seeded with bacterial inoculum as for the classical antibiotic susceptibility testing (Kirby-Bauer method); 5 mm diameter paper filter disks were placed on the seeded medium, at 30 mm distance. Subsequently, the disks were impregnated with 5 μL of the stock solution. The plates were left at room temperature for 20–30 min and then incubated at 37 °C for 24 h. The positive results were read as the occurrence of an inhibition zone of microbial growth around the disk [12–14].

#### 3.2.4. Quantitative Assay of the Antimicrobial Activity

It was performed by binary micro dilution method, in Mueller Hinton broth (MHB) distributed in 96 multi-well plates, in order to establish the minimum inhibitory concentration (MIC) [15–17]. In this purpose, serial binary dilutions of the tested compounds were performed in a 150 μL volume of MHB (from 1000 μg/mL to 7.8 μg/mL) and each well was seeded with 30 μL of microbial inoculum of 0.5 MacFarland density. The plates were incubated for 24 h at 37 °C, and MICs were read as the last concentration of the compound which inhibited the visual microbial growth.

#### 3.2.5. Antibiotic Controls

Beta-lactam antibiotics (*i.e.*, third generation cephalosporin ceftazidime) have been used as controls [15].

## 4. Conclusions

In conclusion, we synthesized some new dibenz[*b*,*e*]oxepin derivatives from the key intermediate 11-hydroximino-6,11-dihydro-dibenz[*b*,*e*]oxepin, which was treated with acyl chloride in order to obtain eight compounds bearing fluorine and trifluoromethyl group substituents in different positions on the phenyl ring. The results of the antibacterial activity of the new compounds indicated that the most active was the 2-F, 5-CF_3_ substituted one, which exhibited good antimicrobial activity against multi-drug resistant strains of *Pseudomonas aeruginosa,* enterobacterial strains and methicillin resistant *S. aureus*, frequently implicated in the etiology of opportunistic infections.

## Figures and Tables

**Table 1:** Data on the new compounds.

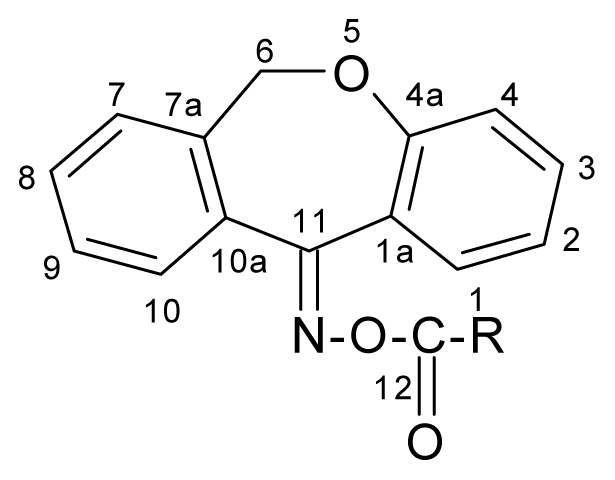									
No.	R	m. p. (°C)	yield (%)	C%		H%		N%	

				c.	e.	c.	e.	c.	e.
a.	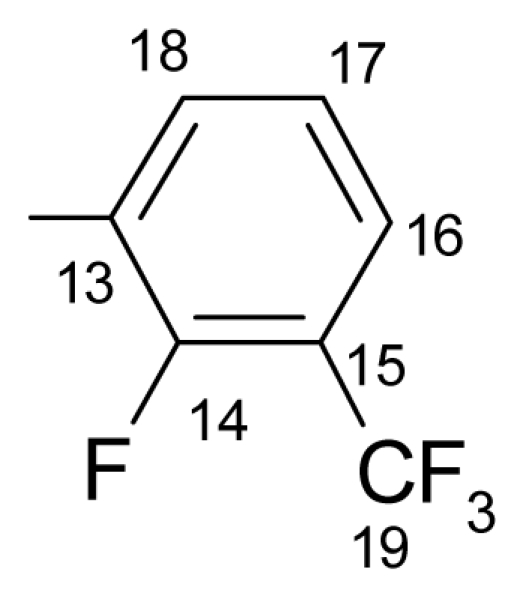	149.9–152.4	68	63.62	63.95	3.15	3.17	3.37	3.41
b.	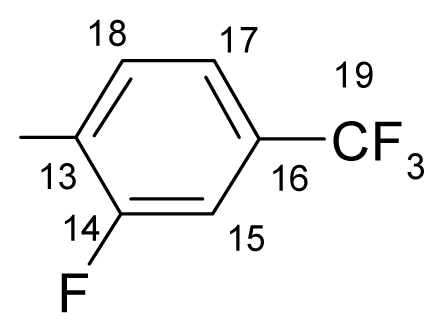	115.6–118.7	74	63.62	63.84	3.15	3.16	3.37	3.31
c.	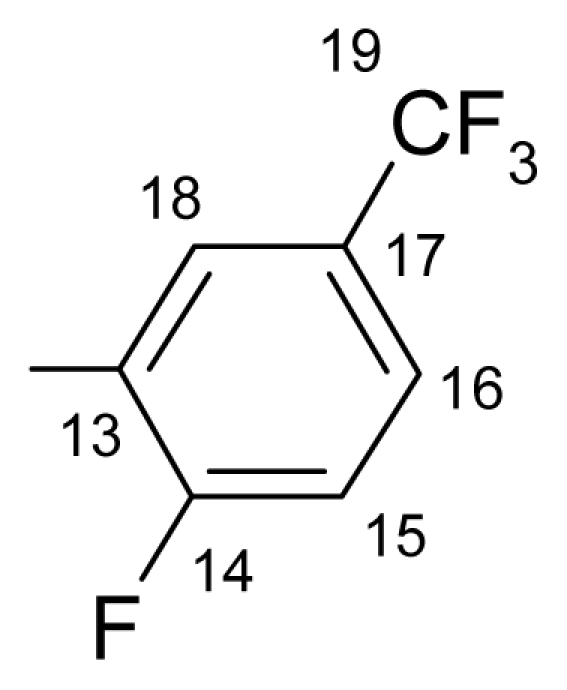	145.1–145.7	78	63.62	63.30	3.15	3.19	3.37	3.39
d.	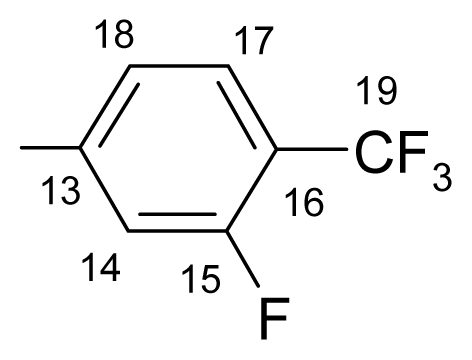	116.5–117.2	82	63.62	63.98	3.15	3.19	3.37	3.31
e.	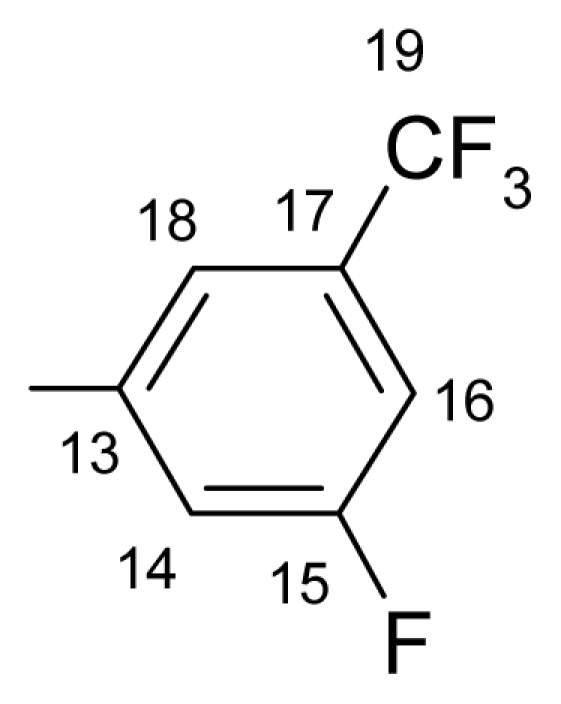	118.4–119.6	66	63.62	63.29	3.15	3.09	3.37	3.36
f.	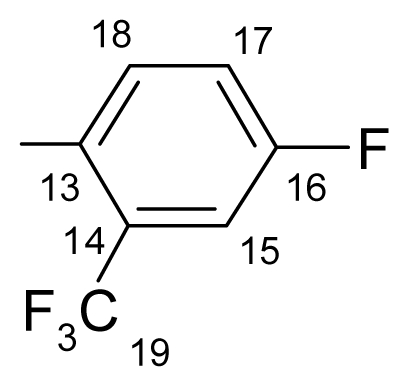	130.4–133.8	86	63.62	63.89	3.15	3.11	3.37	3.35
g.	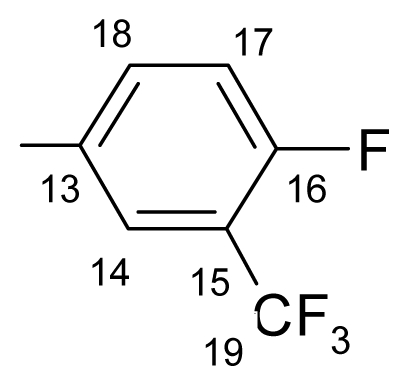	137.5–139.6	68	63.62	63.94	3.15	3.18	3.37	3.31
h.	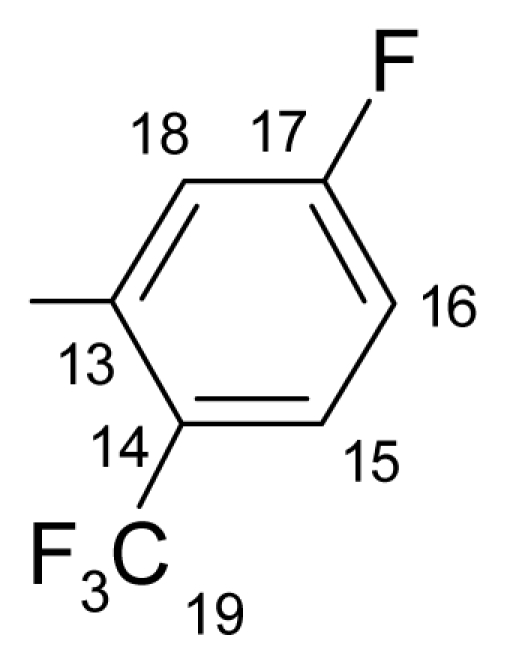	145.3–147.1	62	63.62	63.31	3.15	3.12	3.37	3.39

where: c. = calculated, e. = experimental.
